# Sertoli-Leydig Cell Tumour and* DICER1* Mutation: A Case Report and Review of the Literature

**DOI:** 10.1155/2018/7927362

**Published:** 2018-09-25

**Authors:** B. Wormald, S. Elorbany, H. Hanson, J. W. Williams, S. Heenan, D. P. J. Barton

**Affiliations:** ^1^St George's Hospital, UK; ^2^St George's Hospital and the Royal Marsden Hospital, UK

## Abstract

Sertoli-Leydig cell tumours of the ovary (SLCT) are rare tumours predominantly caused by mutations in the* DICER1* gene. We present a patient with a unilateral SLCT who had an underlying germline* DICER1* gene mutation. We discuss the underlying pathology, risks, and screening opportunities available to those with a mutation in this gene as SLCT is only one of a multitude of other tumours encompassing* DICER1 *syndrome. The condition is inherited in an autosomal dominant fashion. As such, genetic counselling is a key component of the management of women with SLCT.

## 1. Introduction

Sertoli-Leydig cell tumours of the ovary (SLCTs) are a rare type of sex cord-stromal tumour, constituting less than 0.5% of all ovarian cancers. SLCTs contain Sertoli and Leydig cells which are somatic cells found within the male gonad. It is likely that SLCTs derive from primitive pregranulosa cells and therefore represent a pseudo–male gonadal genesis in the ovary. Patients typically present in the 2nd or 3rd decade of life and often have features of androgen excess, such as amenorrhoea, hirsutism, deepening of the voice, and clitoral enlargement. The vast majority, more than 95% of tumours, are unilateral, FIGO Stage 1, and either moderately or poorly differentiated. It is increasingly clear that the pathogenesis of SLCT is through mutation in the* DICER1* gene [1].

## 2. Case Presentation

A 15-year-old girl presented with a background of erratic menstrual periods following menarche at age of 12 years. By first contact she had experienced amenorrhoea for 6 months followed by continuous daily vaginal bleeding for 3 months. She had noticed hair loss, receding hairline, and coarse dark hair on her abdomen, thighs, and bottom. Clinical examination revealed a normally developed female without virilisation of the external genitalia or a change in voice. She was pain free.

Hormone profile revealed raised testosterone (10.1nmol/l Ref: 0.5-3.0 nmol/l), suppressed FSH (<0.1 IU/L Ref: 1-11 iu/L), and borderline SHBG (21 nmol/l Ref: 18 – 114 nmol/L). AFP was raised (137 kU/L Ref: 0-5.8 kU/L) but all other tumour markers, including Beta-HCG and Inhibin, were normal. Urine steroid profile was normal.

**Figure 1:**
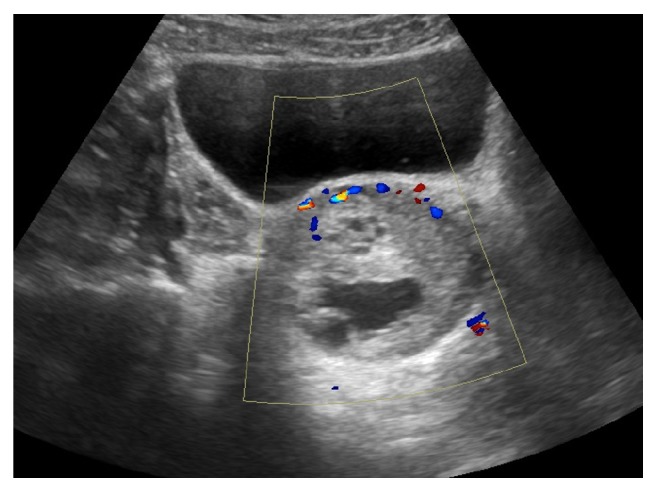
Ultrasound image of ovarian lesion.

**Figure 2:**
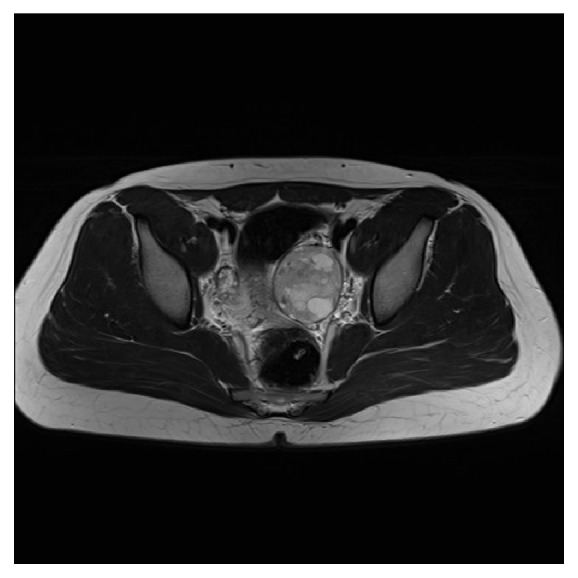
MRI image of ovarian lesion.

Ultrasound examination of the abdomen and pelvis, Figure 1, revealed a complex 7 cm left ovarian lesion with internal vascularity but otherwise normal pelvic organs and adrenal glands. MRI, Figure 2, confirmed an abnormal but well-defined 7 cm left adnexal lesion of predominant intermediate T2 signal interspersed with high signal cystic areas separated by low signal septa. The clinical picture was of a primary ovarian tumour with ectopic production of androgens, and not the more common germ cell tumour.

The case was discussed at the paediatric and gynaecologic oncology MDT. A laparoscopic left oophorectomy with preservation of the ipsilateral fallopian tube was performed with a secondary Pfannenstiel incision used to extract intact the specimen. The tumour which was more solid than cystic was 11 cm in size with no discernible normal ovarian tissue visible. A small nodule on the right ovary was excised. There were no other sites of disease. All other organs and peritoneal surfaces were normal. The postoperative course was uneventful.

**Figure 3:**
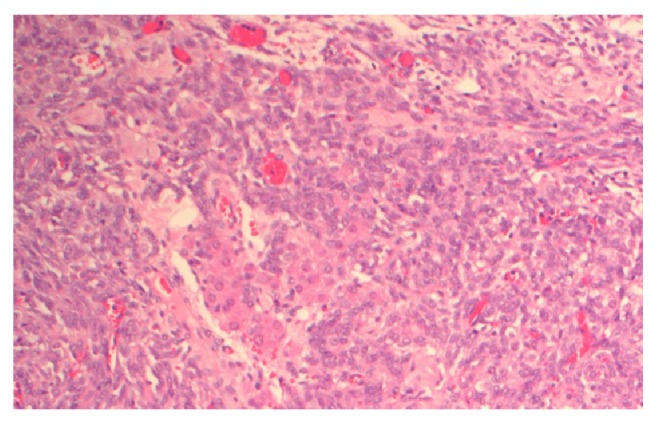
Microscopic view of specimen.

**Figure 4:**
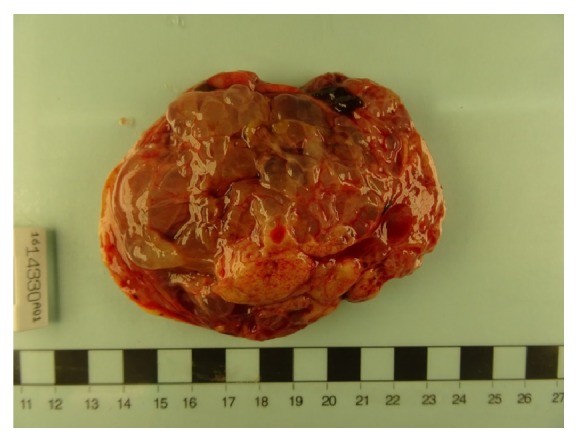
Macroscopic specimen.

Histological analysis, Figures 3 and 4, indicated a predominantly poorly differentiated Sertoli-Leydig cell tumour, retiform pattern, with heterologous mucinous elements. The right ovarian nodule was benign.

Following multidisciplinary team discussion and parental consent, adjuvant chemotherapy was commenced, in a monthly regime of Bleomycin 28500 IU on Day 1, Etoposide 190 mg daily on Days 1-5, and Cisplatin 38 mg daily on Days 1-5 for 3 cycles. Starting prior to chemotherapy commencement, a GNRH analogue, Leuprorelin 3.75mg per month, was administered for 4 months for ovarian protection. The patient became neutropenic following cycle 1 and received Filgrastim 300mcg for 6 days on Days 6-10 of Cycle 2. There were no further episodes of neutropenia. Following cessation of Leuprorelin, menstruation resumed on a regular monthly cycle. She completed her treatment 2 years ago and been reviewed every 3 months. She has had normal tumour markers, including testosterone and AFP, and normal abdominopelvic ultrasound scans throughout this period. Following genetic analysis a germline* DICER1* mutation was discovered, inherited from her father and shared by her 19-year-old sister.

## 3. Discussion

*DICER1* is located on chromosome 14q32.13 and contains 27 exons. Dicer1 is an RNase III endoribonuclease which has several functions but crucially is involved in the microRNA (miRNA) biogenesis pathway. Dicer1 processes precursor miRNA into functional mature miRNA through the cleaving of dsRNA into two RNA strands. RNA IIIa and RNA IIIb domains are responsible for 3p and 5p miRNAs, respectively. The miRNAs act as tumour suppressors in silencing mRNA expression [2].

*DICER1* syndrome is a familial tumour susceptibility syndrome associated with pleuropulmonary blastoma; ovarian sex cord-stromal tumours; cystic nephroma; thyroid gland neoplasia; and other rare benign and malignant tumours. Features of* DICER1* syndrome may present in childhood, but up to 95% of* DICER1* carriers do not develop any significant clinical features by age 10 [3, 4].

*DICER1* related conditions are inherited in an autosomal dominant fashion. Penetrance is currently unknown but thought to be low except patients who have thyroid neoplasia. This is supported by the most recent estimated prevalence of germline* DICER1 *mutation from population databases which is 1:10600 [4].

Germline mutations are typically truncating loss of function mutations. These are mainly single-nucleotide substitutions that produce new stop codons and small insertions or deletions within exons that shift reading frame. The mutations truncate the open reading frame before the end of the RNase IIIb domain, and as such result in complete loss of Dicer protein function [5].

Typically, pathology results from a somatic mutational insult to the remaining wild type allele. This mutation predominantly occurs within exons 24 or 25 in the RNase IIIb domain at one of five hotspot sites (E1705, D1709, E1788, D1810, or E1813) located in the metal-binding site. This affects balance between 5p and 3p miRNA [6].

Previously, up to 60% of SLCT were thought to contain somatic* DICER1* mutations; however, more recently, when centrally reviewed pathology was used to discriminate SLCT pathology, nearly all SLCT contained* DICER1* somatic mutations, especially in those determined to be moderately or poorly differentiated. This is against a background of at least 60% of patients having a germline mutation. Given this, all patients diagnosed with an SLCT should be tested for germline* DICER1* mutation and referred for genetic counselling [7–9].

The effect of this mutation in ovarian tissue includes deregulation of genes that control gonadal differentiation and cell proliferation, downregulation of key ovarian development genes, upregulation of Sertoli cell differentiation genes, and suppression of CYP19A1 leading to reduced aromatase activity causing androgenic effect [10].

### 3.1. Screening

Once* DICER1 *syndrome is identified and the initial neoplasm has been treated, surveillance for recurrence and development of additional* DICER *related tumours should be considered. Genetic counselling regarding the implications of a diagnosis of* DICER1* familial mutation is recommended.

In the case we present the most common residual risk including multinodular goitre and a recurrence or metachronous SLCT. There is also a small increased lifetime risk of other* DICER1* related tumours such as pineoblastoma or cervical embryonal rhabdomyosarcoma. A surveillance program incorporated into the usual ovarian cancer 5-year follow-up protocol would include pelvic ultrasound every 6 months until age of 18, changing to transvaginal approach for better sensitivity in ovarian imaging, and the serum tumour markers AFP and testosterone. Pelvic imaging would be recommended until age 40 to rule out metachronous SLCT, which can be found in 6% of cases up to 14 years following initial development [7]. Symptom awareness for thyroid gland problems is advised, with low threshold for TFTS and thyroid ultrasound. Typically thyroid problems present with palpable neck lumps. The cumulative risk of multinodular goitre or thyroidectomy in women affected by* DICER1* syndrome by age 40 is 75% [11].

Educating those affected to be vigilant of symptoms such as postcoital bleeding, menstrual abnormalities, thyrotoxicity, headache, and visual disturbance and seeking medical review should they occur is also key.

Descendants of those affected have a 50% chance of inheriting the germline mutation and could be offered screening from a much earlier age. This is to incorporate pleuropulmonary blastoma, cystic nephroma, and pineoblastoma and other rarer phenotypes of* DICER1* syndrome.

Screening recommendations in each country are different due to the current evidence base being inconclusive. An international consensus on surveillance guideline is not yet available. However, the following surveillance options have been suggested by teams in Canada and the USA to consider. In the UK, screening is not currently offered but guidelines will be reviewed once international consensus recommendations are published.

Current preliminary recommendations include baseline CT examination of the chest between 3 and 6 months from birth, and if normal, it is repeated when they are 2.5 to 3 years old, sandwiched by chest radiographs every 6 months to age 8 and then annually to age 12.

Abdominal/pelvic ultrasound from birth are repeated every 6 months until age 40. Thyroid palpation annually from age 8 with ultrasound is repeated every 3 years. Brain MRI annually from birth to age 25 is controversial [12, 13].

The risk of repeated ionising radiation exposure is balanced against the early detection of malignant conditions and thus likely improvement in morbidity and mortality. Units have utilised whole-body MRI to ameliorate this; however in very young patients (< 6 years old) the long sequencing time often requires sedation or anaesthesia, which, albeit very low, has its own risk of complications [14, 15].

### 3.2. Conclusion

A diagnosis of Sertoli-Leydig cell tumour should prompt a referral to Clinical Genetics service due to the possibility of germline mutation and familial risk of malignancy. Knowledge of a* DICER1* mutation can inform the individual regarding management of the current tumour and also potential future cancer risks.

## Data Availability

The relevant data to support this case report has been provided in the article. Additional data is not available to protect patient anonymity.
